# Thermosensitive Behavior and Antibacterial Activity of Cotton Fabric Modified with a Chitosan-poly(*N*-isopropylacrylamide) Interpenetrating Polymer Network Hydrogel

**DOI:** 10.3390/polym8040110

**Published:** 2016-03-28

**Authors:** Boxiang Wang, Xiaolin Wu, Jia Li, Xu Hao, Jie Lin, Dehong Cheng, Yanhua Lu

**Affiliations:** 1 Liaoning Provincial Key Laboratory of Functional Textile Materials, Eastern Liaoning University, Dandong 118003, China; bxwang0411@163.com (B.W.); lj18840597623@163.com (Jia L.); cnlnhx@163.com (X.H.); linjieln@163.com (Jie L.); 2 School of Chemical Engineering, Eastern Liaoning University, Dandong 118003, China; wuxiaolin@126.com

**Keywords:** chitosan, interpenetrating polymer network hydrogel, cotton fabric, thermosensitive behavior, antibacterial activity

## Abstract

To increase the themosensitive behavior and antibacterial activity of cotton fabric, a series of poly (*N*-isopropylacrylamide)/chitosan (PNIPAAm/Cs) hydrogels was synthesized by interpenetrating polymer network (IPN) technology using a redox initiator. The IPN PNIPAAm/Cs hydrogel was characterized by Fourier transform infrared spectroscopy (FT-IR), differential scanning calorimetry (DSC), and thermogravimetric analysis (TGA). The results indicated that the IPN PNIPAAm/Cs hydrogel has a lower critical solution temperature (LCST) at 33 °C. The IPN hydrogel was then used to modify cotton fabric using glutaric dialdehyde (GA) as a crosslinking agent following a double-dip-double-nip process. The results demonstrated that the modified cotton fabric showed obvious thermosensitive behavior and antibacterial activity. The contact angle of the modified cotton fabric has a sharp rise around 33 °C, and the modified cotton fabric showed an obvious thermosensitive behavior. The bacterial reduction of modified cotton fabric against *Staphylococcus aureus* (*S. aureus*) and *Escherichia coli* (*E. coli*) were more than 99%. This study presents a valuable route towards smart textiles and their applications in functional clothing.

## 1. Introduction

In the past several decades, smart polymers have been developed to respond to small physical or chemical stimuli such as temperature, pH, ionic strength, and chemical species [1,2] with increasing attention [3]. Of these smart polymers, thermosensitive polymeric materials are considered effective in drug delivery [4,5,6], gene delivery [7], tissue engineering [8], smart textiles fabrication [9], *etc.* Poly(*N*-isopropylacrylamide) (PNIPAAm) is the most intensively investigated thermosensitive polymer. It exhibits a volume phase-transition in response to even slight temperature changes. The coil-to-globule transition, which is a consequence of the rather complex polarity of the molecule occurs at 32 °C—the lower critical solution temperature (LCST) [10]. It is highly responsive to temperature around the physiological temperature due to their unique LCST in the vicinity of human body temperature [11,12]. In addition, the poly(*N*-isopropylacrylamide) has the sharpest phase transition of all thermosensitive *N*-alkylacrylamide polymers [13,14]. Because of this drastic swelling in response to temperature stimuli, PNIPAAm hydrogels has been investigated in the fabrication of smart textiles. For instance, PNIPAAm modified lyocell fibers by graft copolymerization, and the swelling behavior of the poly-NIPAAm/lyocell graft copolymers exhibited a phase transition between 30 and 40 °C [15]. The thermosensitive PNIPAAm brushes were applied to cotton fabric surfaces using a surface-initiated ATRP method, and the surface-grafted PNIPAAm brushes on cotton fabric have a broad transition temperature region below its LCST [16,17].

Chitosan is a naturally occurring polycation that is the second most abundant polysaccharide found on Earth next to cellulose. It is currently manufactured by alkaline de-acetylation of chitin. It has many significant biological and chemical properties—it is biodegradable, biocompatible, bioactive, polycationic, and antibacterial [18,19]. Chitosan is an excellent functional “green” textile finishing agent in the field of textile engineering [20,21]. In terms of textile finishing processes, chitosan can be chemically crosslinked with the textile fabric surface and fixed on the textile surface.

In this study, *N*-isopropylacrylamide (NIPAAm) and chitosan were combined to prepare the intelligent PNIPAAm/Cs hydrogel using an interpenetrating polymer network (IPN) technology. NIPAAm was used as a monomer to synthesize the temperature-sensitive PNPAAm—this is the first network in the IPN hydrogel with an *in situ* method. Chitosan is the second network with PNIPAAm to form IPN hydrogels. It is both thermosensitive and antibacterial. The molecular structure was characterized with Fourier transform infrared spectrum (FT-IR), and the thermosensitive behavior of the IPN hydrogel was investigated with LCST measured by different scanning calorimetry (DSC). The IPN hydrogel was then applied to modify cotton fabric, and the molecular structure and the surface micrograph was characterized with FT-IR and scanning electron microscopy (SEM), respectively. The hydrophobicity, antibacterial activity, and grafting degree of the cotton fabric were studied.

## 2. Experimental Section

### 2.1. Materials

*N*-isopropylacrylamide (NIPAAm) (Aladdin Reagent, Shanghai, China, 98%) was purified by dissolution and recrystallization in hexane. *N*,*N*-methylenebisacrylamide (BIS) (Shanghai Bio Technology Co., Ltd., Shanghai, China, *M*_w_ = 154.17). Glutaric dialdehyde (GA) (25%) and acetic acid (99%) were obtained from Kermel Reagent (Tianjin, China). Chitosan (98% degree of deacetylation), potassium peroxydisulfate (KPS), and sodium hydrogen sulphite (SHS) were obtained from Sinopharm Chemical Reagent Co., Ltd. (Shanghai, China).

### 2.2. Synthesis of the IPN Hydrogel

**Figure 1:**
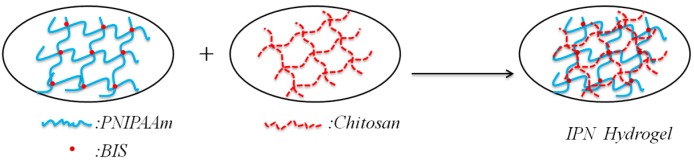
Synthesis scheme of the IPN hydrogel.

The NIPAAm monomer and BIS (as crosslinking agent at 2.0 wt % based on NIPAAm) were dissolved in distilled water to form a monomer solution (6.5 wt %). The 2.5 wt % chitosan acetic acid solution was added and mixed for 30 min. The KPS and SHS served as a redox initiator (2.0 wt % based on NIPAAm). The proportion of oxidant and reducing agent is 1:1. These were mixed in the polymerization solution and stirred for 30 min. The free radical polymerization of the IPN hydrogel was carried out in a glass tube at room temperature (21°C) for 24 h. After polymerization, the synthesized IPN hydrogel was immersed in distilled water at room temperature for 48 h, and the water was refreshed every several hours. The IPN hydrogels were synthesized with feed ratios of NIPAAm and chitosan of 4:1, 2:1, 4:3, and 1:1. The synthesis scheme of the IPN hydrogels is shown in Figure 1.

### 2.3. Grafting of IPN Hydrogel onto Cotton Fabric

**Figure 2:**
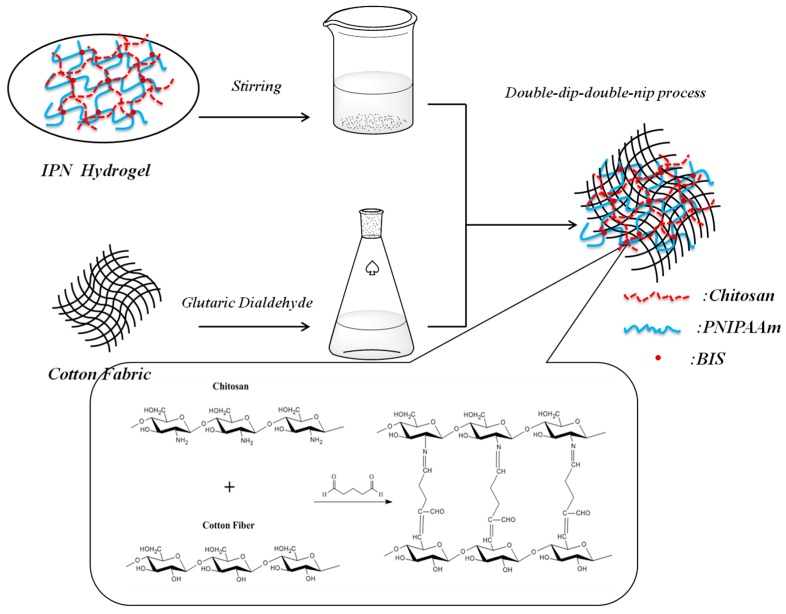
Cross-linking reaction of IPN hydrogel and cotton fabric.

The cotton fabric was washed with acetone, alcohol, and distilled water three times to remove impurities. The pure cotton fabric was then immersed in boiling a 10% sodium carbonate aqueous solution for 2 h, rinsed with distilled water, and dried. Grafting of the IPN hydrogel onto cotton fabric was performed using 5% glutaric dialdehyde (GA) as the crosslinking agent. The cotton fabric was dipped in the IPN hydrogel solution for 30 min with a double-dip-double-nip process. The crosslinking reaction of the IPN hydrogel and cotton fabric is presented in Figure 2. The treatment conditions were as follows: Pre-drying at 60 °C for 5 min, drying at 150 °C for 3 min, washing with distilled water, and vacuum drying at 60 °C overnight.

### 2.4. Analytical Methods

The FT-IR spectra were measured using the Perkin-Elmer Spectrum 100 FT-IR spectrophotometer (Waltham, MA, USA) at room temperature from 4000–500 cm^−1^. Thermogravimetric analysis (TGA) was performed on a Mettler-Toledo TGA/DSC 1 (Greifensee, Switzerland). The surface morphology of cotton fabric samples were characterized by SEM (Zeiss SIGMA, Jane, Germany). The contact angle of cotton fabric was measured by XG-CAMA (Shanghai, China).

### 2.5. Measurement of IPN Hydrogels Thermosensitive Behavior and LCST

The transmittance at 500 nm was measured using 723 UV–Vis spectroscopy. The swelling IPN hydrogel was placed in a 10 mm × 10 mm × 40 mm cuvette. The temperature was raised from 25 to 60 °C, and every test sample was stabilized for 5 min before analysis at that temperature. The LCST was defined as the temperature at the highest point in the derivation curve of transmittance *vs.* temperature [22].

The LCST of the IPN hydrogel sample was determined using a DSC (Mettler-Toledo DSC-822/400). All IPN hydrogel samples were immersed in distilled water at room temperature and swollen to equilibrium before the DSC measurement. Dry nitrogen gas was purged through the DSC furnace at a flow rate of 20 mL/min. The weight of the swollen IPN hydrogel was kept at about 10 mg. Accurately weighed samples were placed in the aluminium sample holder and cooled from ambient temperature to −20 °C. The sample was then heated to 100 °C using an optimum rate of 10 °C/min. The onset point of the endothermal peak was used to determine the LCST [23,24].

### 2.6. Grafting Degree of Cotton Fabric

The cotton grafting degree (*D*_G_) was measured according to a previously reported method [25]:

(1) $$D_{G}\left(\%\right)=\frac{W_{\text{aG}}-W_{\text{aS}}}{W_{\text{aS}}}\times 100$$

Here, *D*_G_ is the degree of grafting (%), and *W*_aG_ and *W*_aS_ are the weights of cotton fabric before and after grafting, respectively.

### 2.7. Measurement of Antimicrobial Activity of IPN Hydrogel Modified Cotton Fabric

Antibacterial activity against *S. aureus* (ATCC 6538) and *E. coli* (ATCC 8099) on cotton fabric before and after modification with the IPN hydrogel were measured according to the Chinese National Standard GB/T 20944.2-2007 that is similar to the ISO/DIS 20743:2005 test method [26,27]. The per cent reduction in the number of colony forming units between the unmodified and modified cotton fabrics after incubation at 37 °C for 24 h was calculated using the following equation:

(2) $$R=\frac{B-A}{B}\times 100\%$$

Here, *R* is the percentage of bacterial reduction, *B* is the number of bacteria colonies (CFU/mL) in the control (unmodified cotton fabric), and *A* is the number of bacteria colonies (CFU/mL) in modified cotton fabric.

## 3. Results and Discussion

### 3.1. TGA and FT-IR Characterization of IPN Hydrogels

Thermogravimetric analysis (TGA) was performed at a heating rate of 10 °C·min^−1^ under nitrogen. The thermal decomposition temperatures and half decomposition temperatures are shown in Table 1. Table 1 indicated that the thermal stability of the IPN hydrogel gradually improved with increasing chitosan content when the feed ratios of NIPAAm and chitosan were below 4:3. The thermal stability of IPN-1 to IPN-4 was all significantly better than that of NIPAAm. The decomposition temperature and half decomposition temperature of IPN-3 were 393 and 429 °C, respectively, when the feed ratio of NIPAAm and chitosan was 4:3. The PNIPAAm began to thermally decompose at 327 °C, and it reached half decomposition at 394 °C. This is attributed to the interpenetrating polymer network technology that made the IPN hydrogel more stable than the general hydrogel because of the more compact structure that resulted from introducing chitosan. However, the thermal stability of IPN-3 is the best when the feed ratio of NIPAAm and chitosan were increased to 1:1.

**Figure 3:**
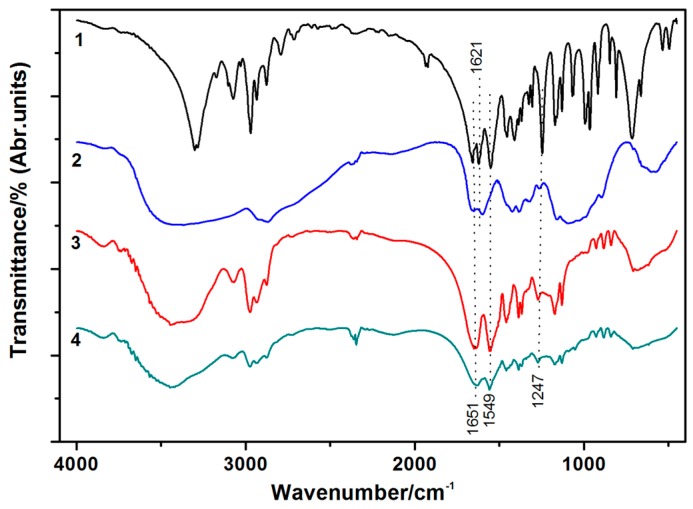
FT-IR spectra of: (**1**) NIPAAm; (**2**) chitosan; (**3**) PNIPAAm; and (**4**) IPN PNIPAAm/CS hydrogel (IPN-3).

The FT-IR spectra of the NIPAAm, chitosan, PNIPAAm, and the IPN PNIPAAm/CS hydrogels are shown in Figure 3. The analysis of the major peaks observed in the FT-IR spectra of the IPN PNIPAAm/CS hydrogel (Figure 3(4)) showed the presence of intense peaks at 1651 cm^−1^ (amide I) corresponding to the C=O of PNIPAAm, 1549 cm^−1^ (amide II) corresponding to the N–H flexural vibration and C–N stretching vibration, 1247 cm^−1^ (C–N–H amide III) corresponding to the C–N–H of PNIPAAm. The disappearance of the 1621 cm^−1^ (C=C) peak indicated that the NIPAAm monomer was successfully polymerized into the products. These spectra revealed that the addition of chitosan had no effect on the positions of the characteristic peaks of the PNIPAAm. This indicated that the chitosan was not chemically bound to the PNIPAAm but was physically interpenetrated within the PNIPAAm hydrogel.

### 3.2. Thermosensitive Behavior of the IPN Hydrogels

#### 3.2.1. Transmittance Measurement of the IPN Hydrogels

**Figure 4:**
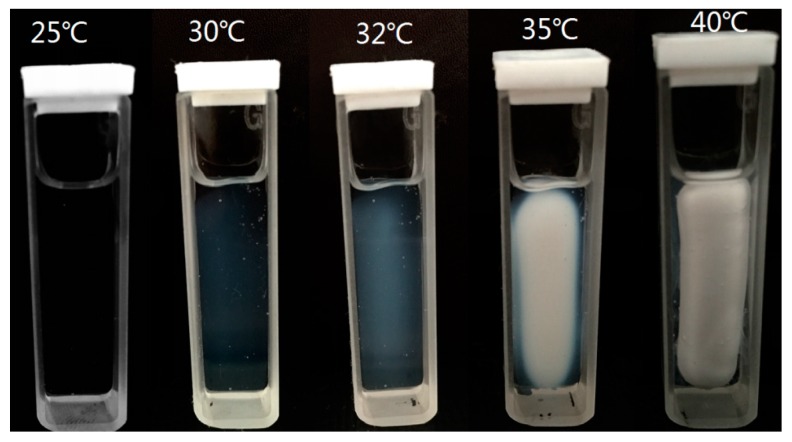
Photograph of IPN hydrogel (IPN-3) at different temperatures.

The thermosensitive behavior of the IPN hydrogel (IPN-3) is shown in Figure 4. The colorless transparent IPN hydrogel gradually becomes white with increasing temperature. The color of IPN hydrogel became quite opaque near 33 °C. Thus, the transmittance of IPN hydrogel sample decreased gradually with increasing temperature. This is a simple method to measure the thermosensitive behavior of the IPN hydrogel.

**Figure 5:**
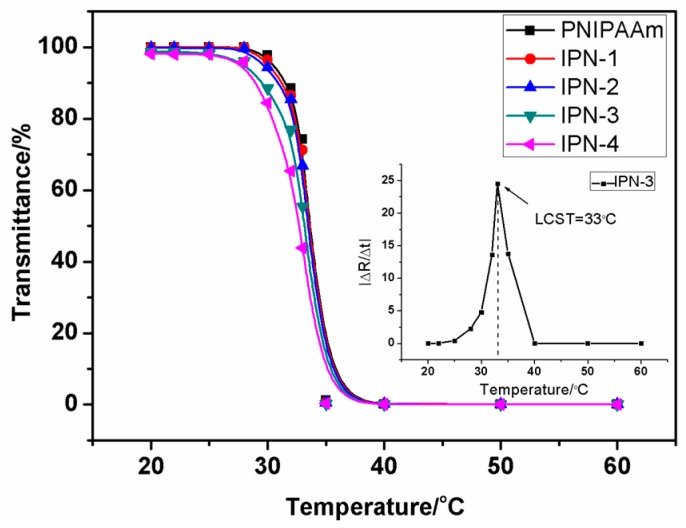
Temperature dependence for the optical transmittance (500 nm) of the hydrogels.

The LCST was defined as the temperature at the highest point of the derivation curve of transmittance *vs.* temperature. The transmittance of each hydrogel sample decreased similarly (Figure 5). The data indicated that the LCST of IPN-3 was 33 °C after curve fitting. All the hydrogel samples had nearly the same LCST of 33 °C. This can be explained in that the chitosan in the IPN hydrogel only acted as the second network and did not react with the *N*-isopropylacrylamide. Thus, the IPN structure had insignificant impacts on LCST as long as both network components within an IPN were chemically identical. 

#### 3.2.2. Determination of IPN Hydrogels’ LCST

**Figure 6:**
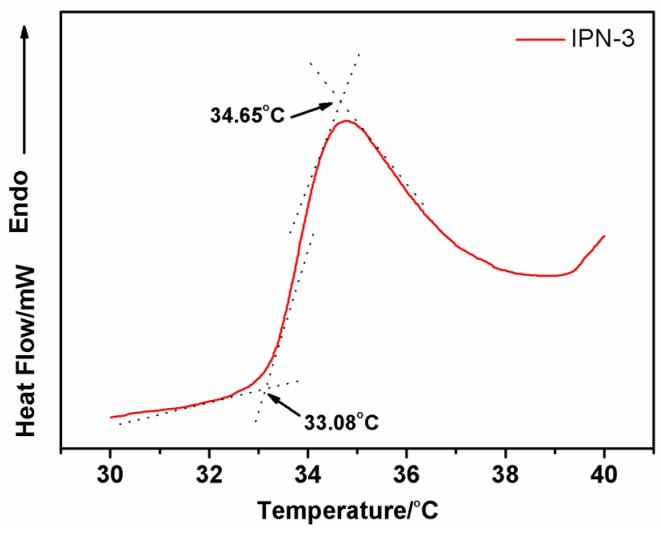
Determination of the LCST from a typical DSC thermogram of the IPN hydrogel.

Poly (*N*-isopropylacrylamide) (PNIPAAm) is one of the most well-known polymers. It exhibits a reversible, temperature-dependent phase transition [28]. Here, the LCST of the IPN hydrogel was accurately determined using DSC. Figure 6 shows DSC curves of the IPN hydrogel (IPN-3). The onset temperature was considered as the LCST value and was determined by the intersection point of the two tangents (Figure 6). The LCST of the IPN-3 in this study was determined to be 33.08 °C. This result was similar to the previous measurement (Figure 5). The peak temperature was close to 34.65 °C—quite similar to human body temperature.

### 3.3. Grafting IPN Hydrogel onto Cotton Fabric

#### 3.3.1. Grafting Degree of Cotton Fabric

There are many –NH_2_ and –OH reactive groups in chitosan in the IPN hydrogel as well as –OH in the cotton fabric. Thus, the grafted reaction could occur between the chitosan and the cotton fabric in the presence of a crosslinking reagent (GA). The grafting degree of the modified cotton fabric is shown in Table 2.

Table 2 shows that the grafting degree of the cotton fabric increased gradually with the increase in chitosan content. There was almost no grafting degree for the PNIPAAm-modified cotton fabric. This is because of the lack of reactive groups to crosslink the polymer. There was a high grafting degree for the IPN hydrogel because those reactive groups in chitosan can react with the cotton fabric. There was no obvious weight loss in the modified cotton fabric after washing five times. This means that the IPN hydrogel was crosslinked to the cotton fabric firmly by covalent bonds. 

#### 3.3.2. FT-IR Analysis of Cotton Fabric

**Figure 7:**
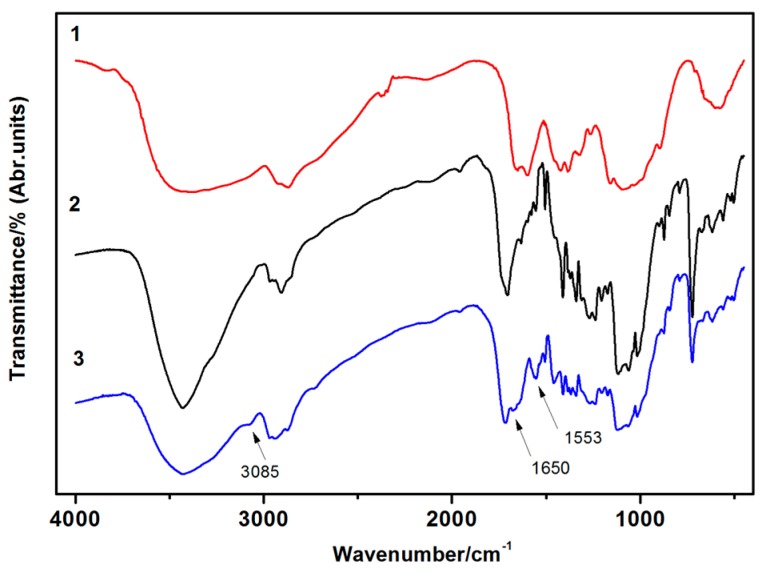
FT-IR spectrum of: (**1**) PNIPAAm/Cs hydrogel; (**2**) cotton fabric; and (**3**) cotton modified with the IPN hydrogel.

The structure of the unmodified and modified cotton fabric was confirmed by FT-IR spectroscopy. The FT-IR spectra of the chitosan, unmodified cotton fabric, and IPN hydrogel-modified cotton fabric are presented in Figure 7, respectively. The analysis of the peaks observed in the FT-IR spectra of the modified cotton fabric showed the presence of an intense peak at 1553 cm^−1^ that could be attributed to the H–C=N group. This proved that the –NH_2_ group on chitosan with GA occurred in the crosslinking reaction and generated a Schiff base. The unsaturated C=C–H stretching vibration of the modified cotton fabric was manifested through the peak at 3085 cm^−1^. This proved that the –OH group on the cotton fabric with glutaric dialdehyde occurred an aldol condensation and generated unsaturated C=C. The double aldehyde group in the GA could react with the cotton fabric and chitosan, respectively. This confirmed that the GA is a “bridge” to link the cotton fabric and the hydrogel. The peak at 1650 cm^−^^1^ corresponding to –NH_2_ groups in the chitosan revealed the connection of chitosan and cotton fabric in the crosslinking reaction of GA. This indicates that the IPN hydrogel can be grafted on and in the cotton fabric because of chitosan and GA. Thus, the hydrophilicity of the cotton fabric changes as a function of the thermosensitive behavior due to IPN hydrogel modification.

#### 3.3.3. SEM Analysis of the Cotton Fabric. 

**Figure 8:**
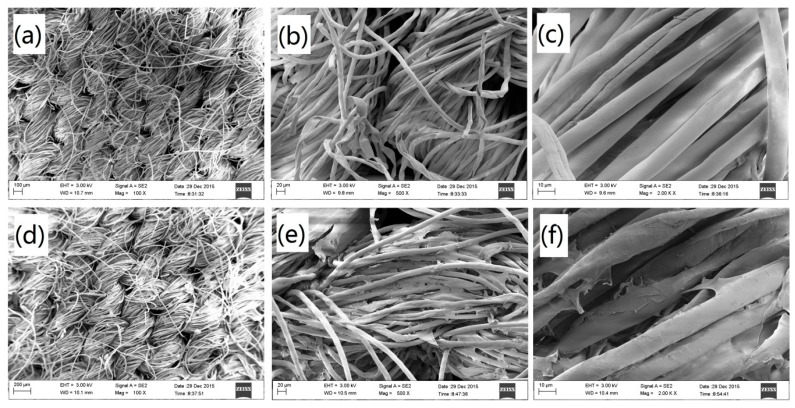
SEM micrographs of unmodified cotton fabric (**a**–**c**); IPN hydrogel modified cotton fabric (**d**–**f**).

Micrograph of the unmodified cotton fabric and the IPN hydrogel-modified cotton fabric was confirmed via scanning electron microscopy (SEM, Figure 8). Figure 8a–c show that the unmodified cotton fabric surface is clean and smooth. Figure 8d–f shows that the modified cotton fabric surface is rough and attached via a layer of the film. This film is homogeneous and is attached to the surface of the cotton fabric with few breaks. This might indicate the presence of the IPN hydrogel coating on the cotton fiber surface. The IPN hydrogel film is distributed well on the surface of the cotton fabric. 

#### 3.3.4. Hydrophobicity of the IPN Hydrogel-Modified Cotton Fabric

**Figure 9:**
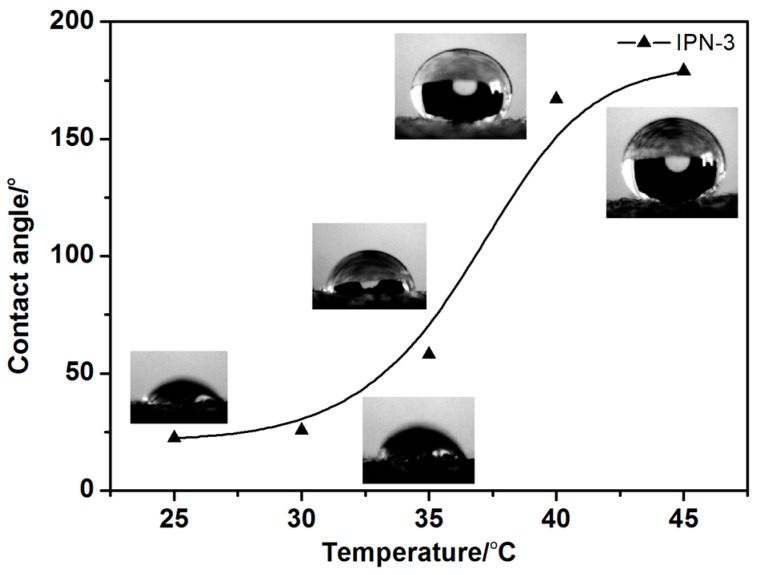
Contact angle measurements of the IPN hydrogel (IPN-3)-modified cotton fabric at different temperatures.

To investigate the thermosensitive behavior of the modified cotton fabric, the hydrophobicity was measured using contact angle measurements of water on the IPN hydrogel (IPN-3) modified cotton fabric. The measurement temperature was 25 to 45 °C. The contact angle measurements of the IPN hydrogel (IPN-3)-modified cotton fabric at different temperatures are shown in Figure 9. The contact angle of the modified cotton fabric increased significantly when the temperature was less than 33 °C (Figure 9). There was a sudden increase when the temperature reached 33 °C. The hydrophilicity of the modified cotton fabric changed with increasing temperature. This sudden change is due in hydrophilicity and hydrophobicity changes on the cotton fabric surface. This change was attributed to the IPN hydrogel on and in the cotton fiber. The sharp increase in temperature around 33 °C in Figure 9 was consistent with the DSC value of 33.08 °C (Figure 6).

#### 3.3.5. Antibacterial Activity of the IPN Hydrogel-Modified Cotton Fabric

To investigate the antibacterial activity of the IPN hydrogel-modified cotton fabric, the bacterial reductions against *E. coli* (ATCC 8099) and *S. aureus* (ATCC 6538) of IPN Hydrogel (IPN-3) modified cotton fiber were evaluated quantitatively in Table 3. This indicated that the bacterial reductions against *E. coli* and *S. aureus* were more than 99%. The high antibacterial activity can be attributed to the following two aspects: (1) The –NH_2_ groups of chitosan in the cotton fabric—confirmed via FT-IR analysis of the cotton fabric modified with the IPN hydrogel (Figure 7)—could contact the bacteria and restrain their free movement. This would inhibit their respiration and eventually cause their death; and (2) the electrostatic attraction between chitosan and bacteria may result in deformation and shrinkage of the cell membranes. This would lead to leakage of the intracellular components including water and protein, and the bacteria would be destroyed [27]. The antibacterial activity is similar to our previous work of chitosan nanoparticles modified with (*A. pernyi*) silk fabric [29]. The activity is higher than that of the chitosan-treated cellulose fabric [30]. Normal cotton fabric has no antibacterial activity but good antibacterial efficacy after modification with IPN hydrogels. 

## 4. Conclusions

The IPN hydrogel was synthesized with NIPAAm and chitosan by the IPN technology. The LCST of the prepared IPN hydrogel was tested at 33 °C, which is similar to human body temperature. The IPN hydrogel was successfully grafted onto the cotton fabric by crosslinking the amino groups of the IPN hydrogel. SEM images confirmed the good combination of IPN hydrogel with cotton fabric. The IPN hydrogel-modified cotton fabric revealed obvious hydrophobicity around the LCST. The IPN hydrogel also provided the cotton fabric with high antibacterial activity.

This study is a first step towards the fabrication of smart textiles consisting of fabric coated with temperature-responsive IPN hydrogels. However, comprehensive phase inversion temperature testing of cotton fabric would be required prior to their use in real applications. Other applications of IPN hydrogel-modified cotton fabrics such as temperature-responsive performance, mechanical properties, dyeing properties, and advanced comfort capabilities will be reported in subsequent manuscripts.

## Figures and Tables

**Table 1:** TGA information of hydrogels.

Samples	Feed ratios of NIPAAm and chitosan	Decomposition temperature	Half decomposition temperature
PNIPAAm	-	327 °C	394 °C
IPN-1	4:1	386 °C	416 °C
IPN-2	2:1	389 °C	424 °C
IPN-3	4:3	393 °C	429 °C
IPN-4	1:1	390 °C	427 °C

**Table 2:** Grafting degree of cotton fabric.

Hydrogel samples	*D*_G_/% (Before washing)	*D*_G_/% (After five times washing)
PNIPAAm	0.7 ± 0.1	0.1 ± 0.1
IPN-1	6.3 ± 0.4	4.9 ± 0.4
IPN-2	7.4 ± 0.4	5.8 ± 0.4
IPN-3	7.6 ± 0.6	6.1 ± 0.6
IPN-4	8.1 ± 0.6	6.6 ± 0.6

**Table 3:** Bacterial reductions of the IPN hydrogel (IPN-3)-modified cotton fabric.

*S. aureus*		*E. coli*	
Control (Unmodified) cotton fabric (cfu/mL)	2.47 × 10^6^	Control (Unmodified) cotton fabric (cfu/mL)	2.34 × 10^6^
Modified cotton fabric (cfu/mL)	0.7 × 10^4^	Modified cotton fabric (cfu/mL)	1.1 × 10^4^
Bacterial reduction (%)	99.72	Bacterial reduction (%)	99.53
